# Ni^2+^ and Cu^2+^ complexes of N-(2,6-dichlorophenyl)-N-mesityl formamidine dithiocarbamate structural and functional properties as CYP3A4 potential substrates

**DOI:** 10.1038/s41598-023-39502-x

**Published:** 2023-08-17

**Authors:** Segun D. Oladipo, Sizwe J. Zamisa, Abosede A. Badeji, Murtala A. Ejalonibu, Adesola A. Adeleke, Isiaka A. Lawal, Amr Henni, Monsurat M. Lawal

**Affiliations:** 1 Department of Chemical Sciences, Olabisi Onabanjo University, P.M.B 2002, Ago-Iwoye, Nigeria; 2 School of Chemistry and Physics, Westville Campus, University of KwaZulu-Natal, Private Bag X54001, Durban, 4000 South Africa; 3 Department of Chemical Sciences, Tai Solarin University of Education, Ogun State, Ijagun, Nigeria; 4 Discipline of Medical Biochemistry, School of Laboratory Medicine and Medical Sciences, University of KwaZulu-Natal, Private Bag X54001, Durban, 4000 South Africa; 5 Faculty of Engineering and Applied Science, University of Regina, 3737 Wascana Parkway, Regina, SK S4S 0A2 Canada

**Keywords:** Cancer therapy, Biochemistry, Biophysics, Cancer, Chemical biology, Computational biology and bioinformatics, Drug discovery, Structural biology, Diseases, Chemistry, Biochemistry, Coordination chemistry, Inorganic chemistry, Materials chemistry, Medicinal chemistry, Physical chemistry, Theoretical chemistry

## Abstract

Metal compounds continued to attract diverse applications due to their malleability in several capacities. In this study, we present our findings on the crystal structures and functional properties of Ni^2+^ and Cu^2+^ complexes of N'-(2,6-dichlorophenyl)-N-mesitylformamidine dithiocarbamate (L) comprising [Ni-(L)_2_] (**1**) and [Cu-(L)_2_] (**2**) with a four-coordinate metal center. We established the two complex structures through ^1^H and ^13^C nuclear magnetic resonance (NMR), elemental, and single-crystal X-ray analysis. The analyses showed that the two complexes are isomorphous, having *P2*_*1*_*/c* as a space group and a unit-cell similarity index (π) of 0.002. The two complexes conform to a distorted square planar geometry around the metal centers. The calculated and experimental data, including bond lengths, angles, and NMR values, are similar. Hirshfeld surface analysis revealed the variational contribution of the different types of intermolecular contacts driven by the crystal lattice of the two solvated complexes. Our knowledge of the potential biological implication of these structures enabled us to probe the compounds as prospective CYP3A4 inhibitors. This approach mimics current trends in pharmaceutical design and biomedicine by incorporating potentially active molecules into various media to predict their biological efficacies. The simulations show appreciable binding of compounds **1** and **2** to CYP3A4 with average interaction energies of –97 and –87 kcal/mol, respectively. The protein attains at least five conformational states in the three studied models using a Gaussian Mixture Model-based clustering and free energy prediction. Electric field analysis shows the crucial residues to substrate binding at the active site, enabling CYP3A4 structure to function prediction. The predicted inhibition with these Ni^2+^ and Cu^2+^ complexes indicates that CYP3A4 overexpression in a diseased state like cancer would reduce, thereby increasing the chemotherapeutic compounds' shelf-lives for adsorption. This multidimensional study addresses various aspects of molecular metal electronics, including their application as substrate-mimicking inhibitors. The outcome would enable further research on bio-metal compounds of critical potential.

## Introduction

Dithiocarbamate usage in coordination chemistry is available in literature^1^ with a lot of structural information for their complexes facilitating numerous applications; it is applicable in photochemistry^2^, catalysis^3^, agriculture^4^, analytical chemistry^5^, and solar energy^6^. It is also employed as a single source precursor to prepare organically caped metal sulfide nanoparticles^7^, inhibitors of cardiac hypertrophy^8^, anticancer candidates^9^, antibacterial compounds^10^, and antioxidant agents^11^. Dithiocarbamates are flexible to functionalize, making it easy to adjust structural architectures and electronic properties^12,13^. A notable synthetic route of dithiocarbamates is the one-pot reaction of the amine and carbon disulfide in a suitable solvent while adding an aqueous metal salt precursor^14^.

Density functional theory (DFT) allows chemical properties calculations to predict the stability, electronic states, and compounds' chemical reactivity, including dithiocarbamate metal complexes^15^. If crystal structures are unavailable, DFT enables geometry and structural elucidation of complexes with their chemical properties^9^. We have previously reported the applications of complexes **1** and **2** as antibacterial and antioxidant agents^16^ with no crystallographic coordinates. Based on our observation, we envisaged that computational studies of the newly reported X-ray crystal structures would provide detailed information on their structural properties, reactivity prediction, and other applications. Therefore, the study aims to provide a detailed structural elucidation of the isomorphous compounds and predict their biological applications theoretically.

With the geometric arrangement of these isostructural moieties and previous knowledge^17^, we envisaged their applications as plausible inhibitors of enzymes causing diseases. This knowledge aligns with an ongoing trend^1,2,8,18^ in therapeutic design and medicine to examine small molecules as potential inhibitors of druggable targets based on their structural details (ligand-based drug design) towards unraveling improved drug candidates. With the moderate biological activity^16^, exploring Ni^2+^ and Cu^2+^ complexes as promising protein inhibitors could be interesting. Cohorts of web-based software serve as pivotal to choosing P450 3A4 (CYP3A4) as the crystalized Ni(II) and Cu(II) complexes' potential target. The cytochrome P450 family is substantially known to participate in phase one metabolism of varieties of endogenous compounds like lipids, bile acids, and steroid hormones. They are responsible for 50% of drug metabolism in humans^19^; a lower level of CYP3A4 would delay several drugs' uptake in the liver and the small intestines. In the liver, CYP3A4 functions as xenobiotic expelling enzymes^17^. Based on cytochrome P450s' essential roles and association with several diseased states, including multidrug resistance in cancer^20^, the search for mitigation is inevitable because cancer is among the dicey and terminal health conditions of equal opportunity. In cancerous cases, this enzyme would eliminate anticancer 'toxic active component,' rendering it ineffective against the cancer cell lines. CYP3A4 overexpression in a diseased state indicates quick metabolism of a therapeutic compound, lowering its retention and shelf-life, thus decreasing the drug's disposition rate to the target.

Protoporphyrin IX, containing Fe (heme), and erythromycin are examples of CYP3A4 substrates. Commercially available potent CYP3A4 inhibitors are clarithromycin, diltiazem, itraconazole, ketoconazole, ritonavir, verapamil, goldenseal, and grapefruit, among others^21^. Previous experimental^22^ and computational^23^ studies showed binding affinities (ΔG_bind_) of sixteen approved inhibitors toward CYP3A4 in the range of –4.09 to –9.96 kcal/mol and 3.46 to –7.94 kcal/mol, respectively. Our latest calculation also showed binding energy (ΔE) values of –15.7, –35.5, –36.4, and –58.1 kcal/mol for copper(II) Schiff-base complexes and heme substrate interaction with the protein^17^. The binding capacities of the known inhibitors depict a relatively lower interaction than the Cu(II) Schiff-base compounds. Therefore, investigations that explore improved CYP3A4 inhibitors are crucial towards moving forward on its biochemical implications to ill-health situations. Hence, we explore the inhibitory properties of Ni(II) and Cu(II) complexes of N-(2,6-dichlorophenyl)-N-mesityl formamidine dithiocarbamate towards CYP3A4 using molecular dynamics (MD) simulations and various analytical schemes to predict improved bioactivity.

## Methods

### Synthesis of [Ni-(L)_2_] (1) and [Cu-(L)_2_] (2)

**Figure 1:**
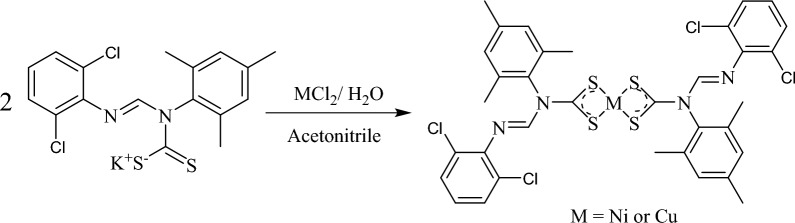
Synthesis protocol of Ni(II) and Cu(II) N-(2,6-dichlorophenyl)-N-mesityl formamidine dithiocarbamate.

The preparation method of complexes **1** and **2** has been reported^16^, with the synthetic route in Fig. 1. The displacement reaction of potassium N'-(2,6-dichlorophenyl)-N-mesityl formamidine dithiocarbamate salt and MCl_2_ (M = Ni or Cu) in a ratio of 2:1 using acetonitrile as solvent produced complexes **1** and **2** in good yield (Fig. 1). The two complexes are thermally stable, intensely colored, and soluble in some solvents like tetrahydrofuran, toluene, dichloromethane, and chloroform, with partial solubility in polar solvents such as ethanol and methanol. Both complexes are stable in air with high decomposition temperatures, ranging between 268–276 °C.

### Single-crystal X-ray diffraction

The apparatus for crystal evaluation and data collection of **1** and **2** is a Bruker Smart *APEXII* diffractometer with Mo Kα radiation (λ = 0.71073 Å) equipped with an Oxford Cryostream low-temperature apparatus operating at 100 K for all samples. We collected reflections at different starting angles and used the *APEXII* program suite for reflection indexing^24^. The process continued with data reduction using the *SAINT* software suite^25^ and the scaling and absorption corrections using the *SADABS* multi-scan technique^26^. The structures were solved by the direct method using the *SHELXS* program and refined using the *SHELXL* program^27^. We used *Olex2* software package^28^ to refine the crystal structures in two stages, including non-hydrogen atoms' isotropic and anisotropic refinement with the full-matrix least square method based on *F*^2^ on *SHELXL*. We positioned all hydrogen atoms (H-atoms) geometrically and unrestricted, allowing the H-atoms to match with their parent atoms and refine them isotropically. The respective dichloromethane and toluene molecules in **1** and **2** become disordered over a distinguished position. In both instances, PART –1 and –2 instructions were used to resolve the disorder, with each component showing 0.5 and 0.125 site occupancy factor in **1**, and 0.25 site occupancy factor in **2**, respectively. The crystallographic data and structure refinement parameters for complexes** 1** and **2** appear in Table 1.

**Table 1:** The summary of X-ray crystal data collection and structure refinement parameters for complexes** 1** and **2.**

	1	2
Empirical formula	C_34_H_30_Cl_4_N_4_NiS_4_^.^1.25CHCl_3_	C_34_H_30_Cl_4_CuN_4_S_4._C_7_H_8_
Molecular weight	972.58 g/mol	920.33 g/mol
Crystal system	Monoclinic	Monoclinic
Space group	*P*2_1_/*c*	*P*2_1_/*c*
a/Å	7.5570(2)	7.5424(8)
b/Å	29.5198(6)	29.800(3)
c/Å	10.2393(2)	10.060(1)
α/°	90	90
β/°	106.398(1)	108.449(2)
γ/°	90	90
Volume/Å^3^	2191.28(9)	2145.0(4)
Z	2	2
ρ_calc_g/cm^3^	1.474	1.425
μ/mm^–1^	1.137	0.988
F(000)	989.0	946.0
Crystal size/mm^3^	0.28 × 0.18 × 0.12	0.26 × 0.15 × 0.11
2Θ range for data collection/°	5.786 to 56	4.482 to 56.384
Index ranges	–7 ≤ h ≤ 9	–4 ≤ h ≤ 9
	–38 ≤ k ≤ 31	–39 ≤ k ≤ 37
	–13 ≤ l ≤ 13	–12 ≤ l ≤ 13
Reflections collected	18,040	12,486
Independent reflections	5222 [R_int_ = 0.0186, R_sigma_ = 0.0202]	5122 [R_int_ = 0.0192, R_sigma_ = 0.0291]
Data/restraints/parameters	5222/86/298	5122/273/303
Goodness-of-fit on F^2^	1.058	1.021
Final R indexes [I > = 2σ (I)]	R_1_ = 0.0391, wR_2_ = 0.0994	R_1_ = 0.0310, wR_2_ = 0.0708
Final R indexes [all data]	R_1_ = 0.0466, wR_2_ = 0.1041	R_1_ = 0.0426, wR_2_ = 0.0751
Largest diff. peak/hole / e Å^–3^	0.97/–0.54	0.41/–0.30

### Theoretical modeling of Ni(II) and Cu(II) complexes

The software packages used for the quantum mechanical modeling of the two structures and calculation setup are ChemDraw^29^ and GaussView 6^30^. We executed calculations on the Lengau cluster of the South Africa Centre for High-Performance Computing [CHPC, www.chpc.ac.za] using Gaussian 16^31^ and AMBER18 GPU-enabled^32^ molecular simulation software package. The theoretical level used in calculating electronic properties of **1** and **2** is M06-L^33^ DFT level with def2-TZVP^34^ basis set. Based on the literature, the selected level of theory is sufficient for the molecular modeling of transition metal complexes^9,33^. Def2-TZVP is a contracted Gaussian basis set that allows scalar relativistic effects approximations in larger atoms^34^. The reliability, time-effectiveness, and accuracy of this basis set and its application to heavier atoms, including metals, have been investigated with various topics^9,15,35–38^. Specifically, we have used the M06-L/def2-TZVP combination to calculate the anticancer properties of gold(III) dithiocarbamate complexes^9^. We predicted the biofunctional implication of these compounds by coupling them with CYP3A4 and performing multiple simulations totaling 1.5 μs, plus various analyses.

#### DFT study of Ni(II) and Cu(II) complexes

We calculated the energy of the frontier molecular orbitals (FMOs), including the highest occupied molecular orbital (E_HOMO_) and the lowest unoccupied molecular orbital (E_LUMO_). The studied quantum chemical descriptors are (equation, Eq. 1–8) ionization potential (IP), electron affinity (EA), band gap (E_g_), chemical hardness (η), global softness (S), chemical potential (µ), electrophilicity index (ω), and electronegativity (χ). Researchers have highlighted the relationships between these molecular orbitals and the selected quantum chemical descriptors in literature^39,40^.

1 $${\text{IP }} = { } - {\text{E}}_{{{\text{HOMO}}}} { }$$

2 $${\text{EA}} = { } - {\text{E}}_{{{\text{LUMO}}}}$$

3 $${\text{E}}_{{\text{g}}} = {\text{E}}_{{{\text{HOMO}}}} - {\text{E}}_{{{\text{LUMO}}}}$$

4 $${\upeta } = \frac{{{\text{E}}_{{{\text{LUMO}}}} - {\text{E}}_{{{\text{HOMO}}}} }}{2}$$

5 $$S = \frac{1}{\upeta }$$

6 $${\upmu } = - \frac{{{\text{IP}} + {\text{EA}}}}{2}$$

7 $${\upomega } = \frac{{{\upmu }^{2} }}{{2{\upeta }}}$$

8 $${\upchi } = { } - {\mu }$$

To estimate electron delocalization, we used the natural bond orbital (NBO) analysis^41^ with the available code in the Gaussian package to better describe the energy-lowering occurrence when the donor and acceptor orbitals mix (Eq. 9)^42^.

9 $$\Delta E_{ij}^{\left( 2 \right)} = \frac{{ - q_{i} \left| {F_{ij} } \right|^{2} }}{{\varepsilon_{j}^{{\left( {NL} \right)}} - \varepsilon_{i}^{\left( L \right)} }}$$

The term $\varepsilon_{j}^{{\left( {NL} \right)}}$ is the energy of the non-Lewis NBO (i.e., π^∗^), $\varepsilon_{i}^{\left( L \right)}$ is the energy of the orbital occupied by a lone pair (n), and q_i_ is the occupancy of the orbital and $\Delta E_{ij}^{\left( 2 \right)}$ is the stabilization energy as determined by second-order perturbation (E^(2)^) treatments.

To estimate the effective atomic charges on the constituting atoms of the complexes, we incorporated Merz-Singh-Kollman (MK)^43^ formalism implemented in Gaussian 16. The MK scheme also estimates the total electrostatic effect from the interaction of the metals with the hybridizing fragments to generate the molecular electrostatic potential (MESP) surface.

#### Molecular dynamics simulations of Ni(II) and Cu(II) complexes with CYP3A4

**Figure 2:**
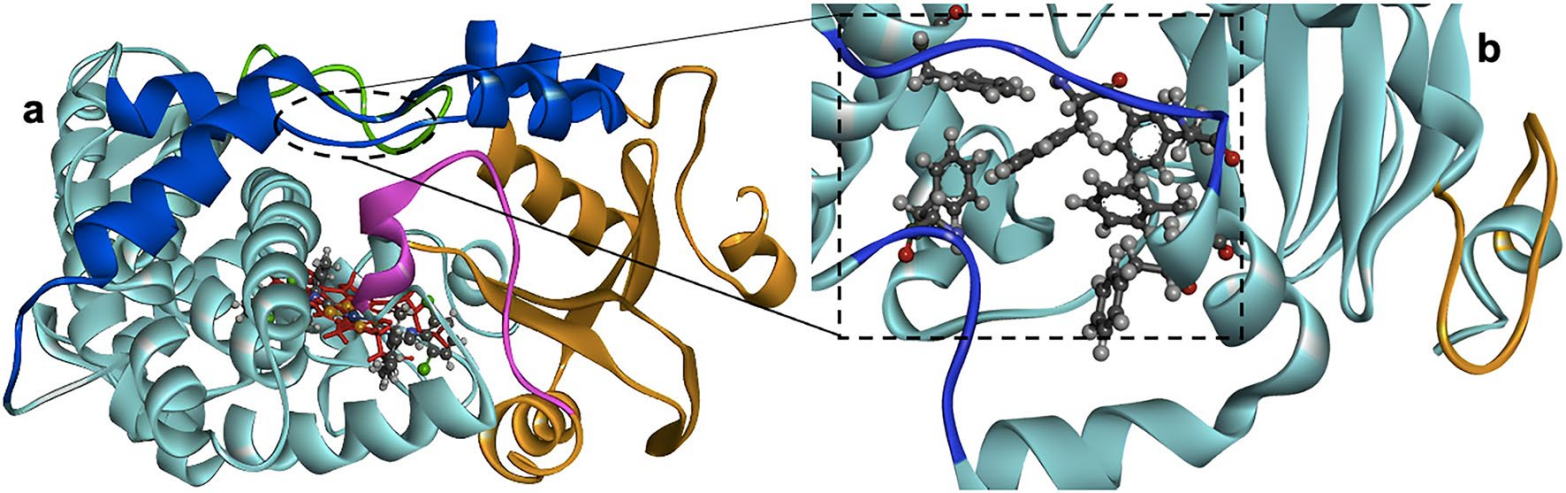
CYP3A4 3D structure showing (**a**) the crucial regions^50^ and overlayed crystal compound 1 (ball and stick model) with the heme (red stick representation) at the substrate binding pocket. The enzyme core (cyan) has several helices, houses substrate binding residues, and interconnects all other domains. The β domain segment (orange; 1–73, 332–359) is a highly mobile scaffold with an open channel between the B–B' loop (magenta) and its β_1_ and β_3_ sheets, facilitating ligand binding to the peripheral binding site. The C-terminal loop appears in green (429–451), and the F, G, F', and G' helices with connecting loops are in blue (174–235). In (**b**), we depict (ball and stick) the unique phenylalanine cluster of Phe185, Phe187, Phe191, Phe192, Phe213, and Phe269^50^. Besides the Phe ensemble, other distinguishing structural properties of CYP3A4 from its isoforms are the N-terminal hydrophobic region (orange) and the truncated F and G helices that form its active site roof consisting of bridging loops (blue) for F and G to the F' and G' helices. *Note*: the sequence numbering is based on the model used in this study.

All-atom simulations allow a detailed understanding of several entities, including singlet ions, monomers, and macromolecules, facilitated by various force field development and implementation^44^. Herein, we applied the ff14SB force field^45^ on the protein and parameterized the metal complexes using quantum mechanics and the MCPB.py code^46^ within the AMBER package. The protein preparation steps entail CYP3A4 retrieval with PDB ID 4D7D^47^, isolation from co-crystalized molecules, and modeling missing loops on the SWISS-MODEL server^48^. We then prepared three protein states consisting of apo and two ligand-bound CYP3A4 of compounds **1** and **2**. The two isostructures present a planar shape like the heme substrate co-crystalized with 4D7D, enabling us to easily superimpose the Ni(II) and Cu(II) complexes at the CYP3A4 active site (Fig. 2) with the Discovery Studio^49^. We prepared three starting structures for each protein model by varying the TIP3P water box size at 8, 10, and 12 Å while neutralizing with appropriate sodium and chloride ions. With the CYP3A4 approximate length of 69 Å, the extended simulation box sizes are significantly wide to prevent the protein from overlapping or interacting with its periodic image in the next cell. The smallest water box of 8 Å on each side of the protein indicates that the solute's periodic images are almost 16 Å far apart.

We simulated all atoms on the GPU version of the Particle mesh Ewald Molecular Dynamics^51^ of the AMBER18 program at two femtoseconds timescale integration. The algorithm used for hydrogen atoms constraint is SHAKE while incorporating Particle mesh Ewald for long-range electrostatic interactions at non-bonded interactions cutoff of 12 Å. We integrated the Monte Carlo barostat for pressure and the Langevin thermostat for temperature control. The simulation process involved several steps of minimization and equilibration before the final production runs. System minimization runs at 2000 steepest descent each, in a ten steps approach of gradual restraint removal to ensure structural integrity. The applied restraint on protein and substrate atoms in the first minimization step was 500 kcal·mol^−1^·Å^−2^. We reduced this restraining force on CYP3A4 and metal complexes in 4 steps 50.0, 10.0, 1.0, and 0.1 kcal·mol^−1^·Å^−2^. Decreasing harmonic restraint on the protein backbone was also in another four stages with the same values, while the tenth minimization step runs with no restriction on any atom. Heating of the solute–solvent proceeds slowly from 0 to 300 K for one ns while exerting 40 kcal·mol^−1^·Å^−2^ force on the Ni(II) and Cu(II) substrate and CYP3A4. Subsequent heating of protein and ligands are in six steps of pressure equilibration at 1 atm with decreasing harmonic restraints 40.0, 20.0, 10.0, 5.0, 1.0, and 0.1 kcal·mol^−1^·Å^−2^ for 200 ps. Finally, we simulated the three protein models of varying solvent sizes in the NPT ensemble for ~ 170 ns to achieve a 1.5 microseconds total production trajectory.

#### Post-simulation analysis

We analyzed the protein motion for the CYP3A4 systems by mapping out the root-mean-square deviation (RMSD) of the backbone atoms unto a two-vector principal component (PC), which enabled distinct conformational states identification using the inflection core state (InfleCS) code^52^. Herein, the RMSD (Eq. 10) of the protein backbone atoms (Cα, N, C) measures the average distance between the trajectories and the starting protein backbone atoms. The v_i_ in Eq. 10 designates the coordinates of the Cα, N, C atoms in v at i time, and w_i_ denotes the coordinates of the Cα, N, C atom in w at the time i – often called cRMSD. The metric used to estimate ligand displacement is distance RMSD (dRMSD); it is the RMSD of all pairs of internal distances between the simulations and the initial structure. The binding affinity of each metal–ligand enclosed by the entire protein scaffold integrates the van der Waals (E_vdW_) and electrostatic energy (E_elect_) components (Eq. 11). The CPPTRAJ^53^ module available in the AMBER suite facilitates linear interaction energy (LIE), RMSD, RMSF, and PC analyses.

10 $$RMSD\left( {v,w} \right) = \sqrt {\frac{1}{n}\mathop \sum \limits_{i = 1}^{n} ||v_{i} - w_{i} } ||^{2}$$

11 $$\Delta E_{int} = E_{elect} - E_{{{\text{vdW}}}}$$

Another dimensionality reduction approach used in this study is linear discriminate analysis (LDA). This Python code Link2 (https://github.com/rnw8253/nsp13_analyses/blob/main/GMM_LDA/gmm_lda/lda.py) enabled cluster prediction and separation of multiple data through single-value decomposition formalism. Westerlund and Delemotte^52^ wrote the InfleCS program, available via GitHub Link3 (https://github.com/anniewesterlund/InfleCS-free-energy-clustering-tutorial) and Link4 (https://github.com/delemottelab/InfleCS-free-energy-clustering-tutorial). The approach integrates Gaussian mixture models (GMM) to construct density that facilitates refined core state extraction. These core states represent metastable conformations within a free energy landscape (FEL) along collective variables (CVs) as expressed in Eq. 12, where $\rho_{a,\mu ,\Sigma } \left( x \right)$ is the Gaussian mixture density at $x$^52^.

12 $$x \in { }{\mathbb{R}}^{Ndims} , G\left( x \right) = - k_{B} Tlog\rho_{a,\mu ,\Sigma } \left( x \right)$$

To predict the structure–function property of CYP3A4 for the complete protein structure during the simulation, we used the TUPÃ module^54^ to estimate the magnitude and direction of electric fields (EF) projected ($\vec{E}$) onto a selected bond axis (Eq. 13). The terms $\vec{E}_{total}$ and $\vec{r}_{bond}$ are the total EF and bond axis vectors, respectively. This metric has facilitated the coupling of electrostatic contributions and protein flexibility simultaneously to describe enzyme catalysis^55^.

13 $$\vec{E}_{proj} = \frac{{\vec{E}_{total} . \vec{r}_{bond} }}{{\left| { \vec{r}_{bond} } \right|}}$$

## Results and discussion

### Spectroscopy studies

We have previously reported the spectroscopy data of **1** and **2**^16^, the azomethine (NC(*H*) = N) protons are the primary diagnostic peak that shifts upfield from 10.11 ppm in **L** to 8.90 ppm in complex **1**. The projections at 2.26 and 6.93–6.98 are assignable to aliphatic and aromatic protons, respectively, while these peaks appear at 2.07–2.23 ppm and 6.81–7.35 ppm in the spectrum of the free ligand. In the ^13^C-NMR spectrum of **1**, the characteristic quaternary thiouride carbon atom peak (–NCS_2_) projects to 218.76 ppm, slightly shifting upfield with 0.28 ppm compared to **L**. The experimental NMR and UV–visible depiction are available in the supporting information, SI (Figures S1 to S3).

FT-IR spectra of** 1** and **2** show four characteristics band of the dithiocarbamate ligand. The first band appears at 1436 cm^–1^ due to the thiouride band υ(N–CS_2_). The second band appears around 1098–1123 cm^–1^ due to υ(C–S) vibrational band. This data indicates the chelating bidentate nature of the dithiocarbamate unit backbone^56^. The other spectra project at 1639–1644 cm^–1^ and 317–354 cm^–1^, attributed to υ(C = Nstr) of the azomethine and metal-to-sulfur bond (M–S), respectively^57,58^. In the electronic spectra of **1**, there are three significant bands at 263, 343, and 465 nm. There are two critical bands at 300 and 449 nm in compound **2**, plus a shoulder band at 321 nm.

### X-ray crystal structures analysis

**Figure 3:**
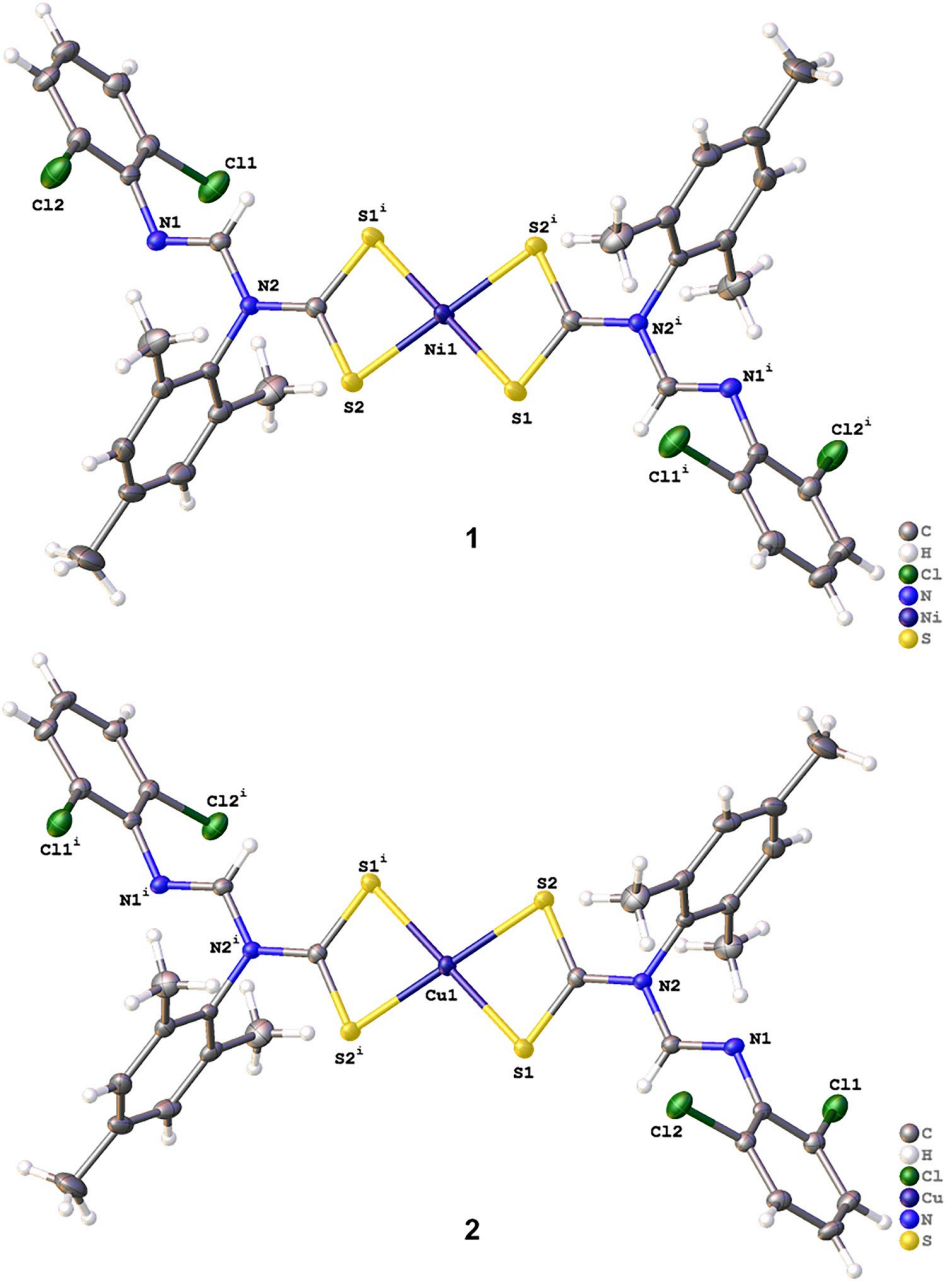
ORTEP diagram for complexes 1 and 2 drawn at 50% thermal ellipsoids probability (we omitted disordered solvent molecules for clarity).

The process of obtaining suitable single crystals is by slow evaporation of dichloromethane and toluene for complexes **1** and **2**, respectively. The structure determination shows that both complexes are monomeric and neutral with the general formula [M(L)_2_] (M = Ni or Cu for **1** or **2**, respectively) with a distorted square planar geometry (Fig. 3). The asymmetric units of both compounds contain half a molecule of the complex on each side. Two pairs of S atoms coordinate the Ni(II) and Cu(II) metal centers on opposite sides while connecting the two bidentate dithiocarbamate ligands. The acute S–M–S bite angles are 79.43° for **1** and 77.54° for **2**, induced by the strained S–C–S bite angles, which distort the geometry around the Ni(II) and Cu(II) ions from perfect square planar. The S–C–S angles are 112.12° and 116.25° for **1** and **2**, respectively. The resulting Ni–S bond lengths in **1** are 2.20 and 2.21 Å, while the Cu–S bonds in **2** are 2.30 and 2.32 Å (Table 2), which agree with values reported for mononuclear dithiocarbamate complexes^59^. The Cu–S bond length is greater than the Ni–S due to differing ionic radii and a lesser force of attraction between electrons and nuclei pull in Cu and S.

**Table 2:** Experimental and calculated (M06-L/def2-TZVP) bond lengths (Å) and angles (**°**) for some selected distances in complexes **1** and **2**.

Parameters	1		2	
	Experimental	Calculated	Experimental	Calculated
Bond length				
M–S	2.20 (4)	2.21	2.30 (5)	2.34
M–S	2.21 (4)	2.23	2.32 (5)	2.35
C–S	1.69 (12)	1.69	1.70 (18)	1.69
C–S	1.71 (18)	1.70	1.71 (18)	1.70
C–N	1.35 (2)	1.36	1.36 (2)	1.37
C–Cl	1.74 (18)	1.74	1.74 (18)	1.73
Bond angles				
S(2)–M–S(1)	79.43 (16)	78.36	77.54 (17)	76.09
S(2)–C–S(1)	112.12 (10)	112.29	116.25 (10)	116.80

The C–N bond length in the –NCS2 fragment of the complexes also deviates from the typical single C–N bond length of 1.47 Å because it is indeed a partial C = N double bond with values 1.35(2) Å and 1.36(2) Å for **1** and **2**, respectively. This result indicates the delocalization of π-electrons over the entire –NCS2 moiety in **1** and **2**^60^. Apart from the bond angles S–M–S with negligible 1.5° difference between experimental and computational output, the structural parameters with M06-L/def2-TZVP calculations are in excellent agreement with the experimental values for complexes **1** and **2** (Table 2). The calculated Ni–S bond lengths are 2.21 and 2.23 Å, Cu–S bond length values are 2.34 and 2.35 Å, while the experimental results are 2.20 and 2.21 Å (complex **1**) with 2.30 and 2.32 Å (complex **2**). Other calculated distances are approximately the same (Table 2). Observing this interrelationship between the computational output and experiment indicates the accuracy of the selected level of theory for the metal complexes. The identical bond lengths and angles in the two complexes from both methodologies buttress their isomorphous relationship.

We further calculated the unit-cell similarity index (π) of complexes **1** and **2** using the Bombicz et al.^61^ formula (Eq. 14) to predict their isomorphous property.

14 $${\uppi } = \frac{a + b + c}{{a^{\prime} + b^{\prime} + c^{\prime}}} - 1$$

Functions a, b, and c and a', b', and c' are the orthogonalized parameters of the two similar structures. The calculated unit-cell similarity index (π) is 0.002; this value is close to zero and implies a significant similarity between **1** and **2**. It is interesting to note that if π = 0, the two structures are predictably isomorphous.

### Hirshfeld surface analysis

**Figure 4:**
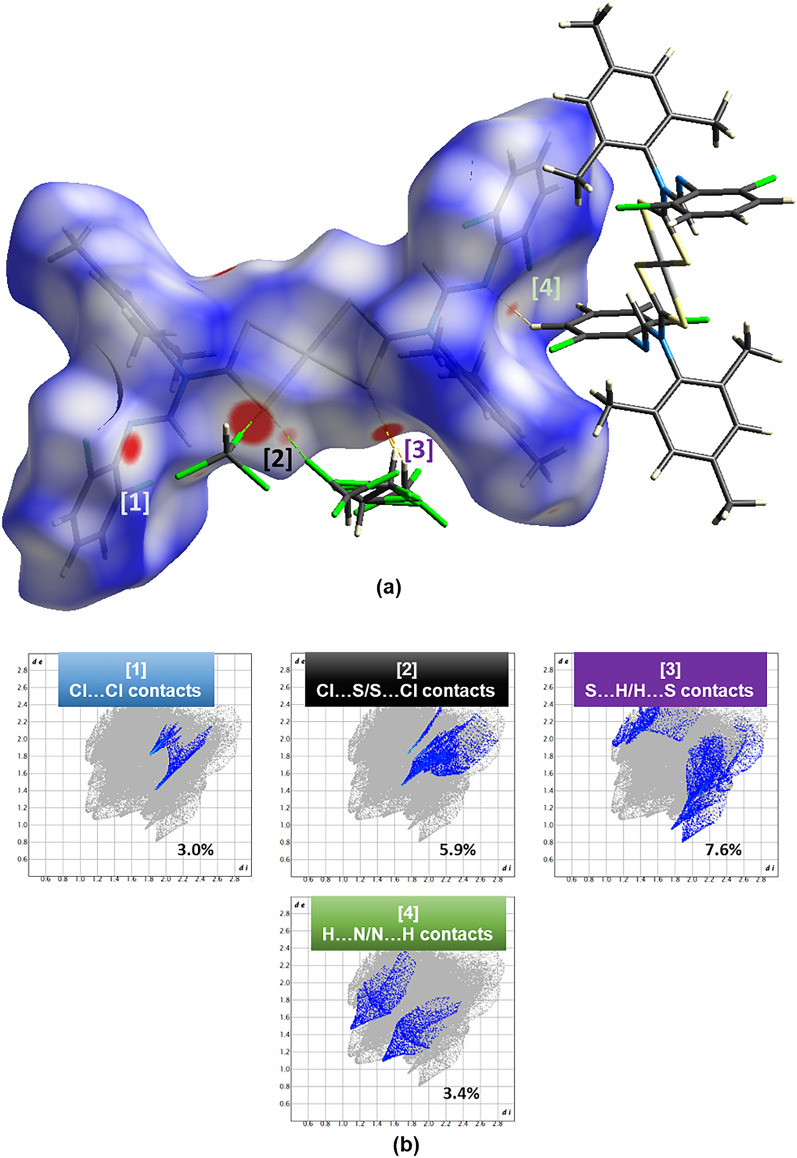
(**a**) *dnorm* property generated over the Hirshfeld surface of** 1** with the short intermolecular contacts (red spots on the isosurface) labeled [1] to [4]. (**b**) The fingerprint plots of the four short intermolecular contacts with their contribution towards the Hirshfeld surface.

**Figure 5:**
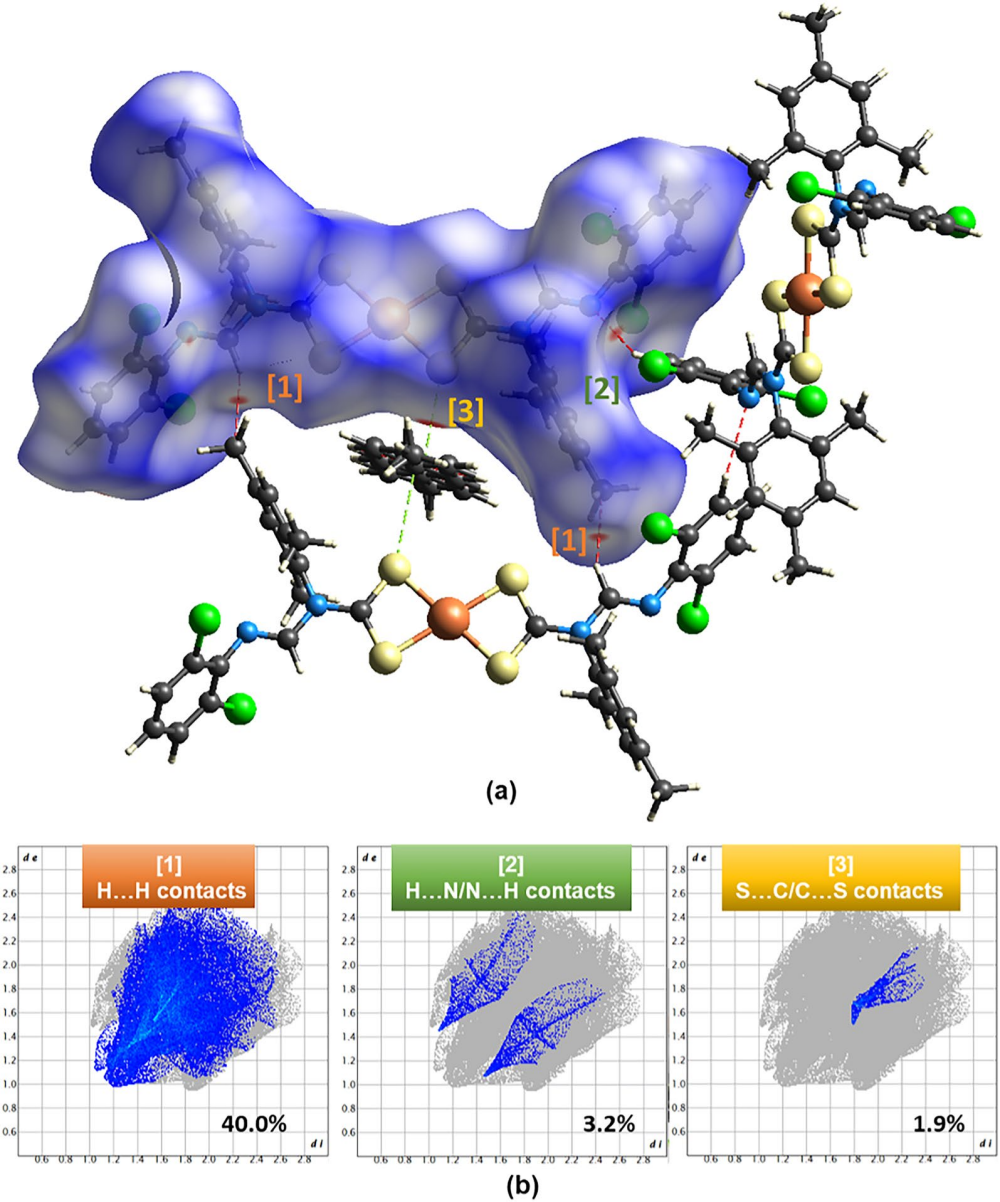
(**a**) *dnorm* property generated over the Hirshfeld surface of **2** with the short intermolecular contacts (red spots on the isosurface) labeled [1] to [3]. (**b**) The fingerprint plots of the three short intermolecular contacts with their contribution towards the Hirshfeld surface.

Hirshfeld surface area is a mathematical concept that defines the surface area of a molecule by dividing it into two regions, namely the inside and the outside. It is obtainable by viewing crystal structures and how molecules interact in their packing systems. Hirshfeld's surface facilitates the exploration of information about all existing weak and strong interactions in the crystal system, which plays a significant role in crystal engineering^62^. In this study, we generate the Hirshfeld surface, mapping with *dnorm* and two-dimensional fingerprint plots, using Crystal Explorer 21^63^ and depict the output in Figs. 4b and 5b. The mapped *dnorm* values^62^ on the Hirshfeld surface appear in red-white-blue color codes, whereby the red regions have negative *dnorm* values, representing short intermolecular contacts; the white has zero *dnorm* values, indicating contact distances relative to van der Waals separation, and the blue regions have positive *dnorm* values, representing farther contacts^64^. It is important to note that we discuss only short molecular contacts in this section since they are preferably significant in contributing to the stabilization of the crystal lattice. Our hypothesis agrees with various studies, including Socha et al*.*'s^65^ works.

The solvent molecules within the crystal lattice of the two complexes drive the variational different types of intermolecular contacts. The chloroform molecule in **1** shows short Cl2•Cl7 (symmetry code: 2–*x*, 2–*y*, 1–*z*; 3.29 Å), S1•Cl5 (symmetry code: *x*, *y*, 1 + *z*; 3.47 Å), and S1•Cl7 (symmetry code: 1–*x*, 2–*y*, 1–*z*; 3.73 Å) contacts, which contribute 3.0%, 5.9%, and 7.6% towards the Hirshfeld surface (Fig. 4a), respectively. The most prominent intramolecular interaction is C18–H18•S2 (symmetry code: *x*, *y*, *z*) and C19–H19•S2 (symmetry code: 1–*x*, 2–*y*, –*z*) hydrogen bonding patterns. The toluene molecule in **2** participates mostly in H•H and S•π_toluene_ intermolecular interactions, contributing 40% and 1.9% toward the Hirshfeld surface, respectively (Fig. 5a). Two intermolecular C–H•N hydrogen bonds appear between the 2,6-dichlorophenyl moiety and the imine nitrogen. These two electrostatic bonds contribute 3.4% and 3.2% (see reciprocal H•N contacts in Figs. 4 and 5) in **1** and **2**, respectively.

We also analyze the energy framework and components for compound **1** at HF/6-31G level of theory. The Crystal Explorer 21^63^ computes the interaction energy between molecule pairs within 3.8 Å contact with the central molecule (Figure S4a) in a symmetric operation (symop) to predict electrostatic, polarization, dispersion, and repulsion energy components (Table S1). The overall values of these energy components are –14.36, –4.57, –68.31, and 31.26 kcal/mol, respectively. This outcome implies that energy contributions from dispersion and coulomb hugely support compound **1** stability. Based on their orientation, the most prominent symop consists of 4 interacting molecular units in the lattice space –x, y + 1/2, –z + 1/2 with a total energy of –16.37 kcal/mol (Table S1). The overall interaction energy between this complex and its neighboring molecules is –53.85 kcal/mol. A summary of the coulomb and dispersion energy components and the total energy is available in Figure S4b–S4d of the SI. All attempts to calculate the energy framework for compound **2** crashed, perhaps due to the required computing power. However, we project that compound **2** will have similar properties to **1** since they are isomorphs of each other. Researchers^66,67^ have used the energy framework approach to analyze the interaction properties of various metal complexes.

### Natural bond orbital (NBO) analysis

**Figure 6:**
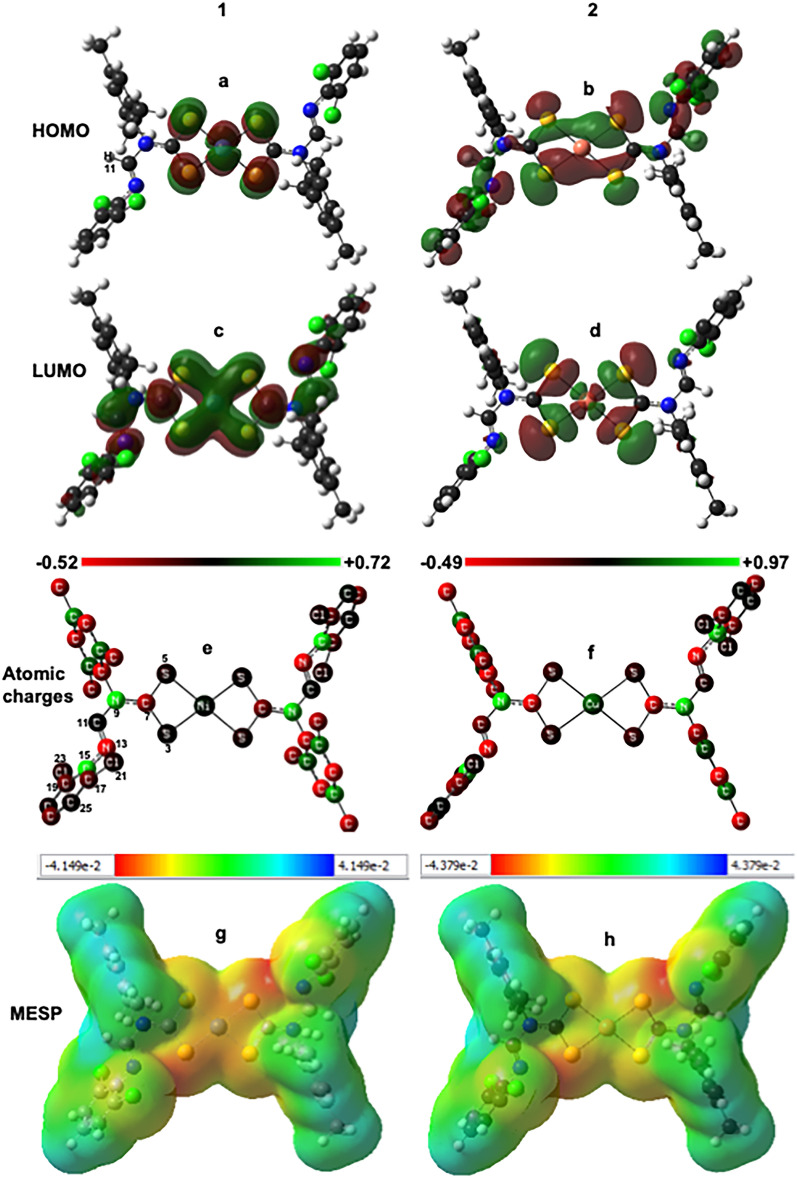
HOMO (**a** and **b**) and LUMO (**c** and **d**) frontier molecular orbitals densities, charge distribution (**e** and **f**) (hydrogen atom hidden for clarity), and MESP surface (**g** and **h**) of isostructural Ni^2+^ (**1**) and Cu^2+^ (**2**) dithiocarbamate complexes at M06-L/def2-TZVP level of theory. The unit of each representation is in atomic units (a. u).

We use the natural bond orbital approach to describe electron delocalization and analyze the stabilization energy per unique donor→acceptor interaction. The stabilization energy, denoted as E^(2)^, is the energy involved in the delocalization of electrons between the filled donor and empty acceptor natural bond orbitals (NBOs)^42,68^. NBO is a quantitative mechanical variable that describes energy-lowering when the donor and acceptor orbitals mix. A large E^(2)^ value permits intense interaction between the electron donors, thereby increasing the extent of conjugation of the entire molecule^42^. Here, we report the calculated donor→acceptor for Lewis-type (Lone-pairs, LP) donor and non-Lewis acceptor interactions with at least 10 kcal/mol E^(2)^ to depict the interactions that contribute considerably to the stability of the complexes. Atom labels and symbols for depiction are available in Fig. 6.

Complex **1** has electron delocalization with high E^(2)^ for N13 → C15–C19 (12.83 kcal/mol), N13 → C11–H11 (13.83 kcal/mol), S3 → N9–C7 (67.54 kcal/mol), S5 → N9–C7 (59.29 kcal/mol), Cl21 → C17–C25 (12.8 kcal/mol), and Cl23 → C15–C19 (12.21 kcal/mol). The nature of these interactions is sigma–pi (σ–π) and π–π, including the lone-pair chloride interactions with triple bonds (one σ and two π bonds). Complex **2** shows the most significant E^(2)^ in S5 → C7–S3 interaction at 30.16 kcal/mol. These intramolecular interactions also occur in the other half of the moiety within these complexes with the same stabilization energies. The predicted energy values for interactions with the filled and empty orbitals are significant to the stability of the complexes. The values in **1** are higher than **2**, denoting that compound **1** is more stable than **2**.

Although complexes **1** and **2** are physically identical, there exist variations in their electronic and atomistic properties. The availability of delocalized electrons with significant E^(2)^ provides more stability to a complex than its counterpart with localized electrons^69^. This observation applies to our present study, whereby complex **1** has appreciable delocalization over **2**. The electronic structure calculation of complex **2** with doublet spin multiplicity (*S* = 1/2) perturbs lower delocalization than **1** with singlet multiplicity. The doublet multiplicity restricted electron mixes within complex **2**, thus decreasing its stability. The NBO analysis enables us to transform the fully delocalized wave function, treating individual chemical bonds and lone pairs distinctively per atom to evaluate the bonding patterns within the complexes. Insight into the molecular and electronic properties of the newly reported X-ray crystal structures facilitates further analysis of the complexes.

### Quantum chemical descriptors of complexes 1 and 2

Analysis of orbital distribution gives insight into how electrons move from occupied to unoccupied orbitals. This modeling provides a fundamental basis for evaluating the compound's chemical reactivity, selectivity, and stability^9^. The reported molecular orbitals are the highest occupied (E_HOMO_) and the lowest unoccupied molecular orbital (E_LUMO_) energies. HOMO and LUMO energies integration facilitate some quantum chemical parameters (Eqs. 1–8) estimation (Table 3). The surfaces of HOMO and LUMO energy distribution in both compounds is available in Fig. 6a–d. With a singly occupied MO at an energy value of –4.06 eV in complex **2**, the probability of forcing an electron to the LUMO under visible light from HOMO decreases, as this SOMO existence slightly slows down this compound's conjugation rate, unlike complex **1** with a shorter HOMO–LUMO gap. A smaller band gap is proportional to a lower UV–Vis frequency, a longer wavelength, and a higher absorption coefficient (Figure S3).

**Table 3:** Quantum chemical descriptors of **1** and **2** at M06-L/def2-TZVP level of theory.

Descriptors	1	2
E_LUMO_ (eV)	–2.69	–2.66
E_HOMO_ (eV)	–4.24	–5.39
Ionization potential, IP (eV)	4.24	5.39
Electron affinity, EA (eV)	2.69	2.66
Band gap, E_g_ (eV)	1.55	2.73
Chemical potential, μ (eV)	–3.46	–4.02
Chemical hardness, η (eV)	0.77	1.37
Global softness, S (eV^–1^)	1.29	0.73
Electrophilicity, ω (eV)	7.76	5.92
Electronegativity, χ (eV^1/2^)	3.46	4.02
Dipole moment (Debye, D)	0.29	1.04

Due to the structural arrangement and electronic distribution of the isostructural complexes Ni^2+^ and Cu^2+^, their charges are 0 with singlet and doublet spin multiplicity, respectively. Complex **2** shows slightly lower LUMO and higher HOMO energy than **1**. These molecular orbitals depict the tendency of a complex to donate its most loosely bound electron (HOMO) to an appropriate orbital of the acceptor molecule (LUMO). Generally, the higher the value of E_HOMO_ of a complex, the better its electron-donating ability, while lower E_LUMO_ favors its electron-accepting ability^70^. Complex** 2** with higher HOMO energy implies a higher electron-donating ability than the Ni(II) counterpart. Also, the values of E_LUMO_ (Table 3) indicate that both complexes are electron acceptors. Lower energy gap E_g_ (E_HOMO_–E_LUMO_) implies high reactivity, with compound **1** showing a lower value than **2**. Also, HOMO and LUMO energies are applicable in predicting inhibition efficiency^71^; the calculated inhibition efficiency is **1** > **2** (from HOMO energy) and **2** > **1**, considering LUMO energy.

The calculated IP values reflect the complexes' nucleophilic properties, while the EA values indicate electron attraction power. Compound **2** has a higher IP value than **1**, inherent from HOMO distribution. Global hardness (η) defines molecules' resistance towards deformation or polarization of the electron cloud under small perturbation of chemical response^72^. The global hardness trend is the same as the energy gap, indicating a marginal difference in both compounds showing approximately 0.8 eV (**1**) and 1.4 eV (**2**). These crystals may not resist deformation or hybridization through coupling with other potentially active moieties. Global softness (S) is the reciprocal of global hardness; the lowest hardness index and highest softness values denote ease of reactivity. The S values of 1.3 and 0.73 eV (Table 3) for the respective complex indicate that compound **1** is softer than **2**, although both compounds are predictably active.

The slight variation in the electronegativity chemical index (χ) of these two complexes (3.46 and 4.02 eV^1/2^) corresponds to the slightly higher dipole moment of complex **2** (1.04 Debye) than the result of **1** (0.26 D). These results indicate that atoms within complex **1** have similar electronegativity values, thereby forming chemical bonds with a lower dipole moment. Authors have associated the electronegativity metric with inhibition efficiency^71^, and our calculation shows that compound **1** will potentially be a better inhibitor than **2**. The calculated electrophilic (ω) properties are 7.76 eV (**1**) and 5.92 (**2**) eV, respectively, indicating compound **1** is more electrophilic than **2**.

Besides the NBO approach, electron densities of atoms from the frontier orbital calculation facilitate the logical characterization of donor–acceptor interactions^73^. Most chemical reactions occur at positions and spatial arrangements where HOMO and LUMO overlap of separated reactants would attain a maximum^74^. The HOMO and LUMO density surfaces (Fig. 6a–d) show that Ni(II) and Cu(II) favorable chelation with the N-(2,6-dichlorophenyl)-N-mesityl formamidine dithiocarbamate occur around the metals–sulfur atoms, which is the region of maximum HOMO and LUMO overlap. Frontier molecular electron densities are metrics to predict drug-receptor interaction sites^75^.

Compound **2** shows a phenomenal singly occupied molecular orbital (SOMO) energy of –4.06 eV (Table 3), denoting the availability of unpaired electrons. From a chemical reactivity perspective, the formation of a transition state occurs during an interaction between the frontier orbitals (HOMO and LUMO) of reacting species^73^. With the presence of SOMO in compound **2**, the ease of FMO transitions (LUMO→HOMO) and optimum interaction would slow down to accommodate the SOMO, thereby slightly distorting the molecular orientation. The only electron transition (S5 → C7–S3) with the most significant stabilization energy (30 kcal/mol) shows a bond angle (S–C–S) in compound **2** with both methods (Table 2). The difference in this angle between both complexes is over 4° (Table 2), buttressing the gentle distortion in Cu(II) compared to Ni(II). Overall, the stability prediction with the NBO method correlates with the results obtained for band energy, softness, global hardness, and dipole moment. Based on these stability indices, compound **1** is presumably more reactive than complex **2**. Therefore, compound **1** would likely interact appreciably with other supramolecules, biological systems, or other molecules in various applications.

### Charge distribution and MESP analysis of Ni(II) and Cu(II) complexes

The population analysis with the MK ESP^43^ approach calculates individual atomic charges per complex (Fig. 6e and f). The charge on each atom maintains intrinsic behavior; for instance, the metals and hydrogen atoms are positively charged in both structures, while all chlorine atoms have slightly negative partial charges. The most significant difference in charge population between the two isomorphs is in the metal centers, where the Ni atom in complex **1** (Fig. 6e) shows a lower (0.047) positive charge value than the Cu atom (0.329) in** 2** (Fig. 6f). Generally, both compounds have various charge distributions, which would facilitate interaction with other moieties. Compound **2**, with a highly positive atomic charge, matches the electronegativity prediction (χ, Table 3).

The drivers of electrostatic interaction are the electrical charges, which have been tagged "crucial" in many chemical reactions and physicochemical components of molecules. Beyond chemical reactivity, understanding charge distribution is among the requirements for detailed molecular behavior prediction, quantitative structural activity relationship, force field development, and drug design^76^. Charge-based descriptors facilitate weak intermolecular interaction estimation, and the electron density from individual atoms within a complex is a reactivity metric. The present population analysis is relevant to the compounds' reactivity prediction. Atoms with negative charge indicate electron availability for positively charged protons attraction. The positively charged projection indicates the availability of more protons than electrons within the atoms (Fig. 6). Therefore, these complexes are not electrically neutral since they do not have an equal number of positive and negative charges. If these complexes interact in a biological medium, they might perturb distinct chemical characteristics.

Figure 6 also shows the electrostatic surface of the isostructural Ni^2+^ and Cu^2+^ complexes. The relative polarity of a molecule is accessible by mapping out its electrostatic net charge surface. The molecular electrostatic potential is crucial to identifying the electrostatic relationship between molecules^77^, and its analysis has been akin to reactivity and binding site prediction^36,75^. Possible sites for electrophilic and nucleophilic attack or hydrogen bonding are predictable through MESP. Therefore, the metric provides an approach to evaluate selectivity and reactivity^78^. The electrostatic potential varies with colors; red shows the region of the most negative electrostatic potential, blue indicates the most positive electrostatic potential, and green is the region of zero potential.

The electron cloud around each atom of both compounds yields all possible electrostatic spectra (Fig. 6g and h). The positive MESP (in a. u) indicates repulsion of the proton by the atomic nuclei in areas with low electron density and partial shielding in the nuclear charge. So, when a positive charge unit moves towards another positive molecular region, there is a repulsive interaction. This repulsion thus gives rise to an increased positive potential energy, and this property appears as blue and light blue color distributions around the complexes (Fig. 6g and h). The red color distribution indicates the availability of nucleophilic region for potential hydrogen bonding, proton attraction, or reaction with positively charged nuclei. The yellow also denotes weak nucleophilic space for reactivity. For instance, the intramolecular interaction of the positive Ni(II) atom with the negatively charged bonded sulfur implies good electron exchange resulting in a stable covalent bond. The electron density isosurface is a surface on which the electron density of a molecule has a precise value that encompasses a given fraction of the molecule's electron probability ρ(r) density. With the isovalue 0.02 a. u. and ρ(r) of 0.0004, the electron densities of the Ni^2+^ (Fig. 6g) and Cu^2+^ (Fig. 6h) complexes have a negligible difference.

### Ni(II) and Cu(II) complexes of N-(2,6-dichlorophenyl)-N-mesityl formamidine dithiocarbamate as feasible CYP3A4 substrates

**Figure 7:**
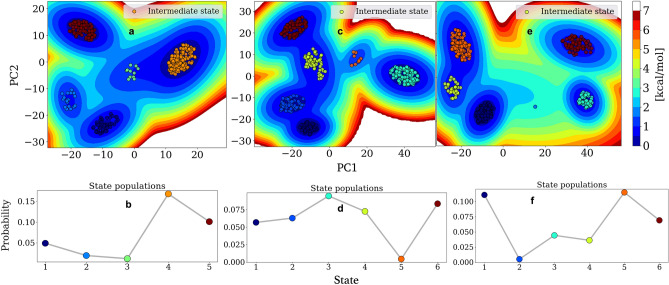
Depiction of the CYP3A4 dynamics. The free energy landscape shows embedded GMM states with the respective population of all identified states for apo CYP3A4 (**a** and **b**), CYP3A4–**1** (**c** and **d**), and CYP3A4–**2** (**e** and **f**). The data are from backbone atoms cRMSD relative to the starting structure, projected onto the PC1/PC2 vectors, and clustered with InfleCS.

The RMSD approach is a long-standing metric for protein dynamics analysis with tenable outcomes. Although the simulation time (0.5 µs per system) is not exceedingly long to trigger large-scale structural dynamics, we expect the metal compounds to perturb substantial changes to the CYP3A4 structure. To estimate CYP3A4 motion and induced changes over the simulation time, we analyze the metastable states obtained by InfleCS based on the principal component analysis (PCA) dimensionality reduction of the protein backbone atoms (Cα, N, and C) RMSD. Figure 7 shows CYP3A4 free energy surfaces with the location of separated states for apo with compounds **1** and **2** substrates bound. The probability density of each state depicts its stability and population likelihood during the simulation.

The unbound CYP3A4 structure samples five states with highly prominent metastable conformations occupying 16.7% and 10% of the distribution (Table 4). The highest stable configuration is also the intermediate structure on the free energy landscape (Fig. 7a and b). The approach to conformer prediction enables us to identify protein states that are often indistinguishable using the RMSD metric of nanoseconds simulations time scale. Ni(II) and Cu(II) substrate-bound trigger protein motion; they sample six different states in total (Table 4). Aside from the 0.4% sampling of protein state 5 (_pro_S5), CYP3A4–**1** substrate complex populates five prominent states within ~ 6 to 9.5% occupancy (Fig. 7c and d). Cu(II) complex at the CYP3A4 substrate site induces six protein states like Ni(II), populating five conformations (Fig. 7e and f) within ~ 3.5 to 11.5% probability of occurrence, and _pro_S2 shows only 0.4% probability.

**Table 4:** Percentage (%) probability of each protein state (_pro_S) identified in PCA and InfleCS clustering of apo CYP3A4, bound Ni(II) and Cu(II) complexes.

System	_pro_S1	_pro_S2	_pro_S3	_pro_S4	_pro_S5	_pro_S6
CYP3A4	4.9	1.9	1.2	16.7	10	–
CYP3A4–**1**	5.7	6.3	9.5	7.3	0.4	8.3
CYP3A4–**2**	11.1	0.5	4.4	3.6	11.5	7.0

Assuming a population state below 3% occurrence is low, there are only three highly sampled states (_pro_S1, _pro_S4, and _pro_S5) in the apo model, while compounds **1** and **2** CYP3A4 systems populate all the protein states with an additional _pro_S6. The most prominent (> 3%) and 'constantly' populated conformation across the three systems are _pro_S1 and _pro_S4. These two states feature unique trends, whereby _pro_S1 shows an increasing order apo < **1** < **2** and _pro_S4 probability shows decreasing values apo > **1** > **2** (Table 4). These contrasting trends are akin to typical substrate conformational selection versus induction processes. Also, _pro_S4 consistently appears as the 'intermediate' state along the energy pathways for all systems to mimic conformation selection and induction on one footing (Fig. 7). This intermediate state could also represent the protein's average structure whose fluctuations drive the CYP3A4 reactivity rate – a dynamic effect phenomenon^55^.

Despite being isomorphous, each metal substrate shows conformational heterogeneity. Ni(II) complex selects and populates an existing lower state _pro_S1, _pro_S2, and _pro_S3 (in the apo form) while redistributing the highly clustered states _pro_S4 and _pro_S5 to induce a new state _pro_S6. Cu(II) complex as CYP3A4 substrate populates _pro_S1, _pro_S3, and _pro_S5 significantly while sharing and inducing an additional state with 7% occupancy (Table 4). With the metal complexes populating _pro_S1 and _pro_S3 moving from unliganded to substrate-bound, we hypothesize a transitioning cycle for the six CYP3A4 states based on the most common state(s) and consistent selection or induction. The proposed free energy pathway between the most common canonical state 4 and substrate-induced state 6 for all the 1.5 µs simulations is _pro_S4→_pro_S1→_pro_S3→_pro_S5→_pro_S2→_pro_S6.

It is interesting to observe total state populations per system as 34.7, 37.5, and 38.1% for apo CYP3A4, CYP3A4–**1**, and CYP3A4–**2**, respectively, implying that substrate-bound proteins did not just 'select' and 'induce' from existing conformers, but explore more trajectories to identify possible heterogeneous conformation(s). The sixth protein state in CYP3A4–metal complexes simulations (at 0.5 µs per system) represents a substrate-induced protein conformation that separates the dynamics of apoenzyme from bound models. This observation differs from an earlier report of CYP3A4 with negligible conformation changes irrespective of substrate/inhibitor interaction over the 1.8 ns simulation^79^. Our analysis shows substrate-dependent conformational dynamics in CYP3A4, consistent with double electron–electron resonance (DEER) and MD study of CYP3A4^80^. Overall, the clustering and free energy method enabled us to uncouple the various core states of CYP3A4 unliganded and liganded structures at a few microseconds time scale.

**Figure 8:**
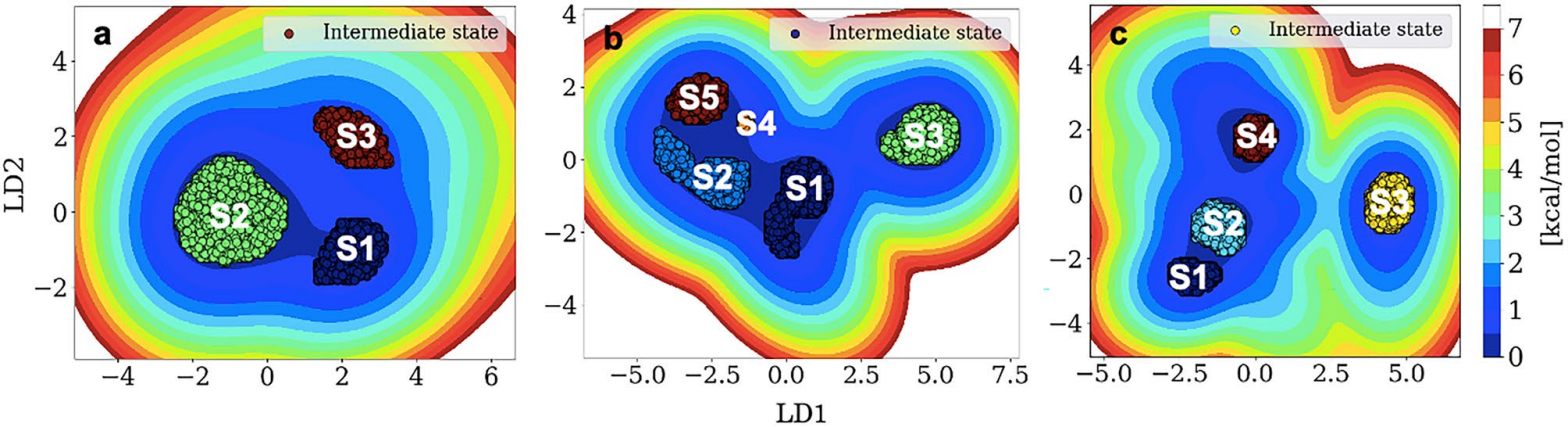
Illustration of CYP3A4 binding site dynamics with LDA dimension trimming and InfleCS clustering. The free energy landscape shows embedded GMM states using multiple distance features constructed from the center of mass separation distance along domains (or loops) crucial to substrate and binding and unbinding of apo CYP3A4 (**a**), CYP3A4–**1** (**b**), and CYP3A4–**2** (**c**). The variables for the distance features are core substrate residues (1); Phe cluster (2); β domain (3); B–B' loop and B' helix (4), yielding six entries. The residues indices are available in Fig. 2, while the selected core residues are 402–405, 408 based on electric field prediction of the most functional substrate residues.

Besides the flat surface area for substrate binding, CYP3A4 has several other binding pockets for ligands, including the established peripheral site above the substrate binding site^81^. We further study Ni(II) and Cu(II) complexes of N-(2,6-dichlorophenyl)-N-mesityl formamidine dithiocarbamate as potent substrates of CYP3A4 by estimating the active region plasticity (Fig. 8). The analysis entails clustering (on two LD vectors) the center of mass interdomain distances between core residues–Phe cluster, core residues–β domain, core residues–B–B' loop/B' helix, Phe cluster–β domain, Phe clusters–B–B' loop/B' helix, and B–B' loop/B' helix–β domain. The selected mobile regions are essential to substrate or ligand binding and unbinding to CYP3A4^17^. This analysis enables us to easily account for substrate cleft motion and flexibility irrespective of ligand presence.

Metastable states identified through protein backbone analysis show that complexes **1** and **2** trigger a unique conformational ensemble absent in the apo CYP3A4. So, we envisaged significant states from CYP3A4 mobile regions when substrate bound or in free form. Figure 8a shows the apo simulation populating three core states on the free energy landscape with S3 as the intermediate conformation and S2 as the most populated configuration (Table 5). The identified conformations depict a few canonical GMM states from the mobile CYP3A4 regions. Although our starting protein structure co-crystalizes with a substrate–ligand complex^47^, it is fascinating to note the differential dynamics of the binding groove in the apo model compared to the metal compounds bound.

**Table 5:** Probability of the states for substrate (S) groove motion sampled by each system. These states were determined by LDA dimension reduction of interdomain distance features and InfleCS clustering and state predictions for apo CYP3A4, bound Ni(II) compound, and Cu(II) complex.

System	S1	S2	S3	S4	S5
CYP3A4	0.07	0.34	0.02	–	–
CYP3A4–**1**	0.1	0.1	0.1	0.003	0.06
CYP3A4–**2**	0.04	0.1	0.06	0.08	–

The apo binding site conformational ensemble appears in triangular connectivity concatenated in a 'well' surface density to depict the compactness of a previously widened CYP3A4 binding groove. CYP3A4–**1** and CYP3A4–**2** simulations populate five protein structural flexibility states, selecting and perturbing two additional substrate site states to the existing three from the unliganded model. Assuming a probability below two decimal places is low, CYP3A4–**1** and CYP3A4–**2** systems populate S5 and S4 (Fig. 8b and c), respectively, to uncouple the heterogeneous state of substrate-induced conformations versus uninhibited structure. This analysis also implies enhanced CYP3A4 active site mobility by substrate docking despite sitting flat in the protein core.

To explore the effect of compounds **1** and **2** on the entire structural components, we measure all atomic fluctuations on a per-residue basis. Analysis of the protein structural flexibility per residue with root-mean-square fluctuation (RMSF) shows average mobility of 14.7 ± 4.5 Å, 12.5 ± 3.4 Å, and 14.6 ± 4.2 for apo, Ni(II), and Cu(II) bound CYP3A4 systems (SI, Figure S5). This result implicates a tiny protein structural fluctuation with substrate interaction compared to its apo model. The less significant effect of substrate presence on the total structural integrity of CYP3A4 potentially indicates its selectivity for the metal compounds in a competitive binding mechanism. Our prediction correlates with ligand-based changes in the CYP3A4 conformational mobility^80^, whereby substrate/inhibitor integration facilitates better stability on the active region dynamics (Fig. 8) than the entire structure (Figure S5). This active region multistate and dynamism would enhance CYP3A4 plasticity and selectivity for several substrates and ligands with easy product release^80^.

Although we include all distance features signatory to the overall binding surface conformational states, substrate entry/binding would likely not involve the Phenyl cluster that is distal from the active site. Therefore, we show in Table 6 the most vital protein structural distances per predicted binding protein states. The flexibility of the β domain and B–B' loop with the B' helix facilitates substrate binding^82^ on the core loop. The catalytic site flexibility measured with interdomain distances (Table 6) shows data ranging from 16 to 26.7 Å. We calculate the interdomain distances per-state basis to estimate if the identified conformations significantly vary. The output shows the substrate-induced states (S4 and S5) populate longer interdomain distances than S1 to S3, denoting CYP3A4 binding region widening as the protein transits from apo to bound tightly and loosely. Based on the active site dynamics (Fig. 8), enzyme–substrate conformation selection, and substrate-induced conformation (Table 5), mobile domain flexibility (Table 6), we propose an energy pathway for the binding site conformations. Considering a 'closed' to 'open' CYP3A4 active site dynamics, S1–S3 mimics a closed state, S4 would be a semi-closed configuration, while S5 presents a hypothetical open state. These canonical states illustrate varying protein-substrate conformation during the binding to fit in the active pocket, bind tightly, and dissociate^80^.

**Table 6:** Average and standard deviation values (Å) of interdomain distances crucial to Ni(II) and Cu(II) substrates' stabilization for each binding site state in CYP3A4 systems.

Distance	S1	S2	S3	S4	S5
Core residues–B–B' loop and B' helix	17.2 ± 1.8	17.4 ± 1.7	16.1 ± 0.3	18.7 ± 0.7	20.4 ± 0.8
B–B' loop and B' helix–β domain	19.7 ± 0.4	19.4 ± 0.5	20 ± 0.8	19.2 ± 0.02	20.2 ± 0.6
Core residues–β domain	24.5 ± 1.6	24.2 ± 1.9	24.6 ± 1.8	26 ± 0.3	26.7 ± 1.5
Total average	20.5 ± 3.5	20.3 ± 3.3	20.3 ± 3.8	21.3 ± 3.7	22.4 ± 3.7

**Figure 9:**
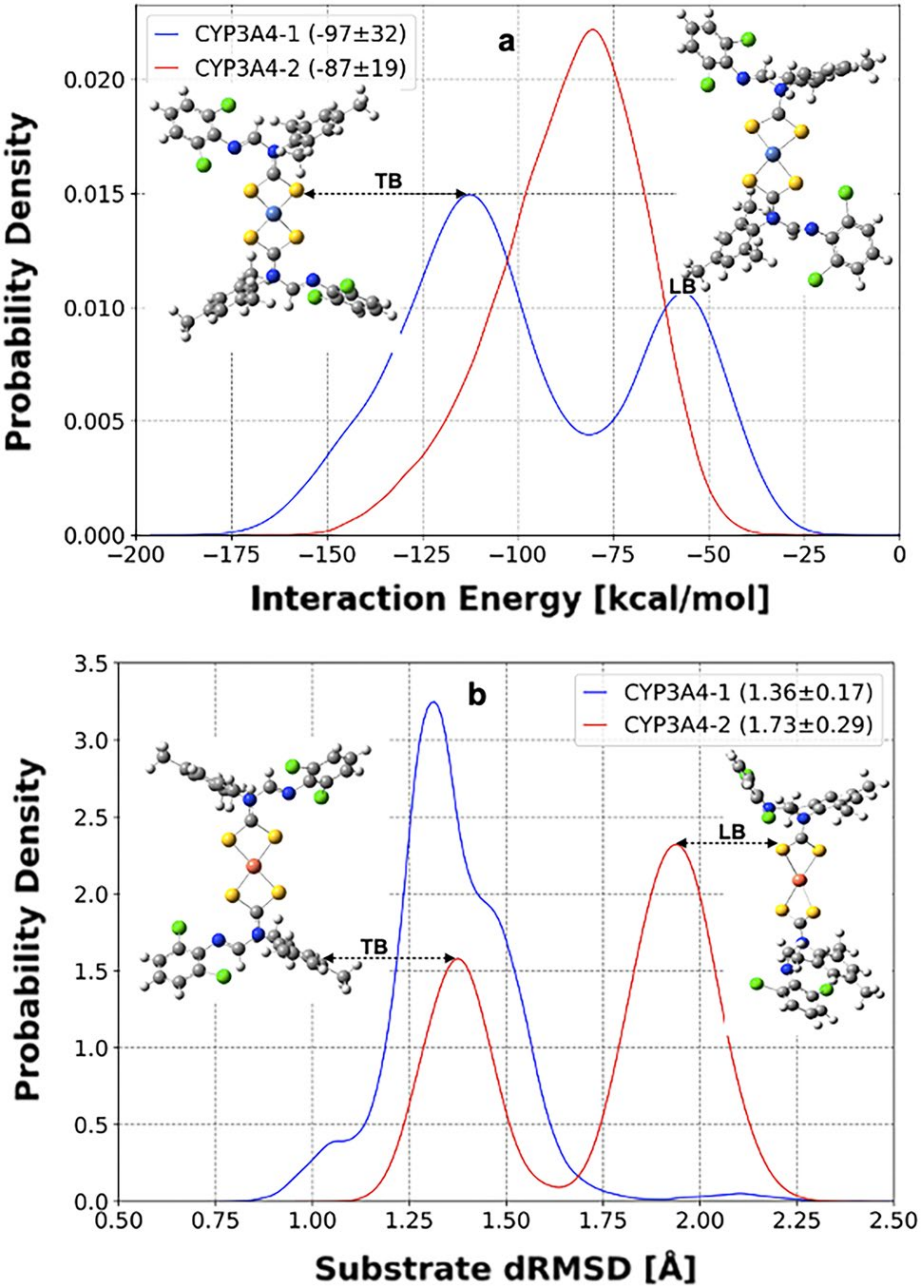
Interaction energy profile (**a**) and depiction of conformational orientation with distance RMSD relative to starting structure (**b**) of Ni(II) and Cu(II) complexes of N-(2,6-dichlorophenyl)-N-mesityl formamidine dithiocarbamate at CYP3A4 substrate region. Average and standard deviation values are in brackets with representative structures for tightly bound (TB) and loosely bound (LB).

Beyond loop motions and improved conformational ensembles of CYP3A4–**1** and CYP3A4–**2** simulations, Ni(II) and Cu(II) dithiocarbamate would undergo conformational changes to fit, bind, or unbind at the substrate site. Although substrate binding tremendously impacts the active region dynamics, the total electrostatic effect propagated from the entire protein scaffold is critical to supporting substrate stability and reactivity^55^. This perception buttresses a report of several CYP3A4 established inhibitor interactions in a model system with calculated binding energy (ΔE) from –0.92 to –17.71 kcal/mol despite integrating the heme natural substrate^23^. Estimating functionality by protein fragmentation and clustering with ligand-substrate seldom yields accurate mechanistic depiction, especially with protein structures of diverse, active regions like CYP isoforms. Figure 9a shows the calculated interaction energy of Ni(II) and Cu(II) compounds within the entire CYP3A4 structure. Compound **1** simulation populates broader protein interaction (–9 to –185 kcal/mol) than compound **2** (–26 to –167 kcal/mol) with average values –97(32) kcal/mol in **1** and –87(19) kcal/mol in **2**. The highly exergonic calculated ΔE values reflect appreciable binding and stability of both compounds with the CYP3A4 structure.

Visualization of simulated trajectories shows no diffusion of Ni(II) and Cu(II) dithiocarbamate out of the active site. Therefore, the potent substrates maintained a bound conformation throughout the simulation while populating various conformers. An inspection of two representative structures at both energy ends (Fig. 9a) shows a highly ordered orientation versus slightly strained square planarity of compound **1**, which we label as tightly bound (TB) and loosely bound (LB), respectively. This observation supports an induced fit binding mechanism whereby a reorientation from the optimum enzyme–substrate geometry causes poor interaction. In summary, both isomorphous compounds show possibilities as CYP3A4 substrates with more promising interaction than the heme substrate with a binding energy of –36.4 kcal/mol^17^. The calculated binding energies are also higher than inhibition binding free energy (estimated from IC_50_), ranging from –4.09 to –9.96 kcal/mol for 16 known inhibitors of CYP3A4^22^. The studied metal compounds have antibacterial properties^16^, thus buttressing metal-based compounds wide range of applications, including therapeutic and pharmacologic^83^.

Structural dynamics are essential to bioactivity and ligand stability prediction^84,85^. Besides highlighting the chemical properties of compounds **1** and **2** in isolation using DFT calculations, we attempt to explore their behavior within a biological media over the simulation time. Figure 9b summarizes the distance RMSD of Ni(II) and Cu(II) complexes relative to their starting geometries. CYP3A4–metal simulations populate Ni(II) structure in an approximate unimodal distribution, while compound **2** shows a distinct bimodal distribution. Despite being isomorphous, the two metal ligands have differential dynamics when incorporated with the same biomolecule. The respective average dRMSD values of **1** and **2** are 1.36 and 1.73 Å, denoting more displacement or deviation of compound **2** from its starting structural geometry arrangement than **1**. Although this analysis intends to address Ni(II) and Cu(II) substrate dynamics during CYP3A4 binding, this structural distortion could also be a latent limitation of the metal center parametrization. However, it is interesting to note that our electronic calculation prediction of better reactivity and stability of compound **1** than **2** corresponds to the structural dynamics of both complexes. As shown in Fig. 9b, the LB representing structure has its Cu–S bonds stretched, distorting the square planar center compared to the TB model.

**Figure 10:**
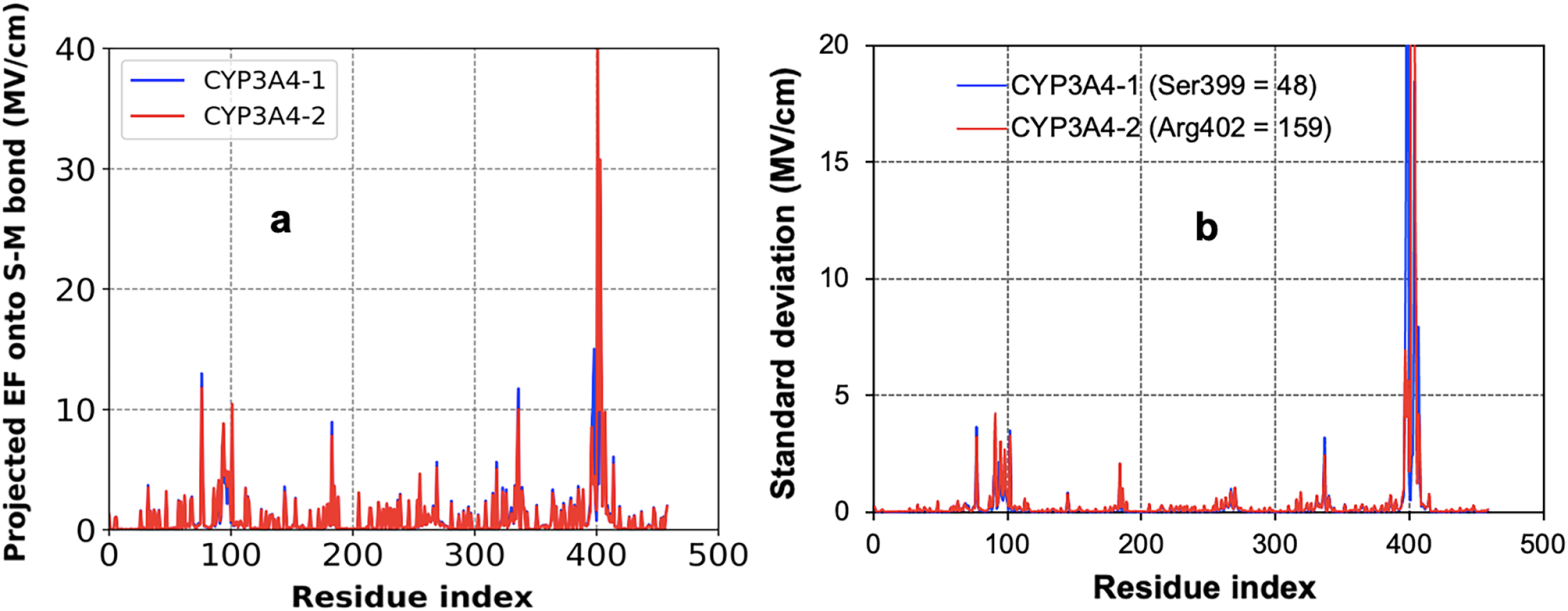
Average (**a**) and standard deviation (**b**, values above 20 are in brackets) of the projected electric field, EF (in MV/cm) per residue onto bond Cys404(S)–Ni(II) in **1** and Cys404(S)–Cu(II) in **2** for CYP3A4 function prediction.

We attempt to account for structural dynamics and electrostatic properties of the protein complexes to predict CYP3A4 function using a single metric that entails calculating the driving force facilitating all motion of electrons in the protein structure. The decomposed projected EF per molecular contributions as a function of time and space $\vec{r}$ (Eq. 13) enabled averaging over per residue output while probing the S–M bond (Fig. 10). The selected query is vital to all cytochrome P450 function (inhibition and catalysis) prediction^50,79^ involving a water molecule and side chain atom (S) of Cys404 (in this case) stabilizing the substrate metal (M) center at the catalytic region. The mechanism is reversible since the substrate is not covalently bound to the enzyme, with S–M non-bonded interaction maintained throughout the catalysis cycle. Therefore, our simulated electric field with the substrate-bound trajectories facilitates combined electrostatic preorganization and structural dynamics measurement.

The output electric field per residue is positive, indicating force direction on the positively charged Cys404(S)–M(II) interaction as electric field direction. So, the EF along this space orients radially outward to indicate residues–substrate bond formation necessary for optimum bioactivity. The quantity of force per unit charge exerted on Cys404(S)–M(II) bond by each residue is almost indistinguishable in both metal compounds' protein models (Fig. 10), with the highest projection from Arg402 at 40 MV/cm for CYP3A4–**2** system. Using electric field values (Fig. 10a) above 7.5 MV/cm as highly significant, eight residues consistently appear in the compound **1** and **2** bound CYP3A4 simulations (Table 7). Arg77 and 337 belong to the B–B' loop/B' helix and β domain, respectively, denoting their importance to substrate binding and catalysis. This observation corresponds to the analysis of crucial domains in the substrate region (Table 6). Several active site residues in the CYP3A4 core are fundamental to its function (Table 7), including Gly398 and Ser399 with respective EF values of 10 MV/cm and 15 MV/cm in the Cys404(S)–Ni(II) direction. Asn403 has a significant (10 MV/cm) projected EF along Cys404(S)–Cu(II) bond.

**Table 7:** Highest and common average electric field (MV/cm) per residue projected along Cys404(S)–Ni(II) in **1** and Cys404(S)–Cu(II) in **2** bonds for CYP3A4 substrate binding.

Residue index	CYP3A4–**1**	CYP3A4–**2**
Arg77 (B–B' loop and B' helix)	13	11.8
Thr102 (Core)	9.5	10.4
Arg184 (Core)	8.9	7.8
Arg337 (Beta domain)	11.7	10
Phe397 (Core)	8.1	8.5
Cys404 (Core)	26.6	30.7
Ile405 (Core)	13.8	14.1
Arg408 (Core)	9.4	9.8

The total higher EF projections per residue in CYP3A4–**2** system (513 MV/cm) than CYP3A4–**1** (488 MV/cm) is like the structural flexibility data with respective average RMSF values of 14.7 Å versus 12.5 Å (Figure S5). Although not huge, the EF (even RMSF) outcome indicates that compound **1** slightly restricts CYP3A4 molecular mobility more than complex **2**. The catalytic residues seem more involved with the binding process (electrostatic interaction) than the protein scaffold dynamics in CYP3A4–**1** system, corresponding to the improved interaction energy profile (Fig. 9a). To verify this guess, we report the deviations in the electric field projections over the 1 μs timescale of the substrate-bound conformational states (Fig. 10).

Interestingly, the highly contributing residues to electrostatic interactions of Ni(II) and Cu(II) complexes at the CYP3A4 catalytic active site (Fig. 10a) are also the most flexible (Fig. 10b). Arg402 residue at the protein's active site has the highest electrostatic contribution and shows an extremely high flexibility of approximately 159 MV/cm in CYP3A4–**2** simulations. This highly significant fluctuation could be from the various rotamers (due to its flexible sidechain) attained from this Arginine during the simulation. Our hypothesis agrees with an earlier structural depiction of this residue's varying orientation linked to its potential flexibility at the CYP3A4 active region^86^. Ser399, with the second highest EF (15 MV/cm), has the most fluctuating residue (48 MV/cm) in CYP3A4–**1** simulation. This outcome mimics a connection between the electrostatic component and structural dynamics of the CYP3A4 structure, whereby the residues interacting with compounds at the active region are also the highly flexible residues in all the conformational ensembles^55^. Although several studies have featured CYP3A4 structural components with essential functional residue identification, our analysis with EF provides an improved perspective. Understanding the prominent residues for substrate binding/catalysis is a prospect in the molecular modeling of drugs and inhibitors.

## Conclusion

This work features a theoretical approach to gain insight into the structural and electronic properties of the two newly reported X-ray crystal structures of nickel, Ni(II) and copper, Cu(II) complexes of N'-(2,6-dichlorophenyl)-N-mesityl formamidine dithiocarbamate. The crystal structures have the Ni(II) and Cu(II) metal centers bonded by four sulfur atoms (from the two dithiocarbamate ligands), forming bidentate coordination directly with the metals. The resulting complexes conform to distorted square planar geometries. The chemical properties prediction with DFT showed that Ni(II) complex **1** is more reactive and stable than Cu(II) compound **2**. Hirshfeld surface analysis showed that the solvent molecules contributed to the intermolecular contacts of both compounds in the crystal lattice. Reactivity prediction through several metrics enabled us to probe the integration of these complexes in a unique biomolecule (CYP3A4) using MD simulations and several analytical metrics. The apoprotein and its bound models populate several conformational states in a selective and inductive mechanism. Determination of the essential domains that support CYP3A4 substrate site plasticity enables us to measure the stability of the Ni(II) and Cu(II) complexes when coupled to the protein. The average binding energy values of –97 and –87 kcal/mol for compounds **1** and **2** in CYP3A4 reflect favorable interaction compared to its heme substrate (–36.4 kcal/mol^17^). Electric field calculation enabled us to capture the electrostatic effect generated from the rigid preorganized protein scaffold as a driver of substrate binding and catalysis. Elucidating the structure–function properties with EF facilitates essential residues' identification whereby the most flexible CYP3A4 residues are also the highly contributing entities to the electrostatic interaction of compounds **1** and **2** at the catalytic interface. Taking together the various experiments, the crystalized isomorphous Ni(II) and Cu(II) complexes of N'-(2,6-dichlorophenyl)-N-mesityl formamidine dithiocarbamate are promising substrates of CYP3A4. Our results could guide further substrate-based and structure-based inhibitor design for cytochrome P450 isoforms.

### Supplementary Information

Supplementary Information.

## Data Availability

CCDC 1997207 and CCDC 1997208 contain supplementary crystallographic data for complexes **1** and **2**. These data are available free of charge with the links 1997207 (https://www.ccdc.cam.ac.uk/structures/Search?Ccdcid=1997207&DatabaseToSearch=CSD) and 1997208 (https://www.ccdc.cam.ac.uk/structures/Search?Ccdcid=1997208&DatabaseToSearch=CSD) or from the Cambridge Crystallographic Data Centre, 12 Union Road, Cambridge CB2 1EZ, UK; fax: (+ 44)1223–336-033; or via e-mail: deposit@ccdc.cam.ac.uk. All data generated or analyzed in this study are reported in this published article and its supplementary information files.
