# Supplementary material for: Ni2+ and Cu2+ complexes of N-(2,6-dichlorophenyl)-N-mesityl formamidine dithiocarbamate structural and functional properties as CYP3A4 potential substrates
Source: Sci Rep. 2023 Aug 17;13:13414. doi: 10.1038/s41598-023-39502-x (PMC10435461; doi:10.1038/s41598-023-39502-x)
Supplement: Supplementary file 1 — Supplementary Information. [file 41598_2023_39502_MOESM1_ESM.docx]

Supporting Information

for

Ni^2+^ and Cu^2+^ complexes of N-(2,6-dichlorophenyl)-N-mesityl formamidine dithiocarbamate structural and functional properties as CYP3A4 potential substrates

Segun D. Oladipo,^a,b^* Sizwe J. Zamisa,^b^ Abosede A. Badeji,^c^ Murtala A. Ejalonibu,^d^ Adesola A. Adeleke,^a^ Isiaka A. Lawal,^e^ Amr Henni,^e^ and Monsurat M. Lawal.^d^*****

^a^ Department of Chemical Sciences, Olabisi Onabanjo University, P.M.B 2002, Ago-Iwoye, Nigeria.

^b^ School of Chemistry and Physics, Westville Campus, University of KwaZulu-Natal, Private Bag X54001, Durban 4000, South Africa.

^c^ Department of Chemical Sciences, Tai Solarin University of Education, Ijagun, Ogun State, Nigeria

^d^ Discipline of Medical Biochemistry, School of Laboratory Medicine and Medical Sciences, University of KwaZulu-Natal, Private Bag X54001, Durban, 4000, South Africa.

^e^ Faculty of Engineering and Applied Science, University of Regina 3737 Wascana Parkway, Regina, Saskatchewan Canada S4S 0A2

***Corresponding authors**: Dr S. D. Oladipo ([segun.oladipo@oouagoiwoye.edu.ng](mailto:segun.oladipo@oouagoiwoye.edu.ng)) and Dr M. M. Lawal ([lawalmonsurat635@gmail.com](mailto:lawalmonsurat635@gmail.com))

# Supplementary data

CCDC 1997207 and CCDC 1997208 contain supplementary crystallographic data for complexes **1** and **2**. These data can be obtained free of charge via <http://www.ccdc.cam.ac.uk/conts/retrieving.html>, or from the Cambridge Crystallographic Data Centre, 12 Union Road, Cambridge CB2 1EZ, UK; fax: (+44)1223-336-033; or via e-mail: [deposit@ccdc.cam.ac.uk](mailto:deposit@ccdc.cam.ac.uk). Experimental NMR and UV-vis spectroscopy data plus the calculated RMSF plot are available herein.

**Figure S1**: ^1^H-NMR spectra of complex **1**

**Figure S2**: ^13^C-NMR spectra of complex **1**

**Figure S3**: Experimental (**a** and **b**) and calculated (**c** and **d**) UV-visible spectrum of complex **1** and **2.**

**Figure S4**: Energy frameworks depiction for compound **1**. (**a**) Color coding (**Table S1**) of neighboring molecules around the central molecule (gray) for the various interacting molecular pairs. Various energy components electrostatic (**b**), dispersion (**c**), and total energy (**d**) predicted through the energy frameworks network using 300 tube size for the visualization.

**Table S1**: Molecular pairs and the interaction energies (kcal/mol) obtained at HF/6-31G level from energy framework calculation for compound **1**.

|  | **N** | **Symop** | **R** | **E_ele** | **E_pol** | **E_dis** | **E_rep** | **E_tot** |
| --- | --- | --- | --- | --- | --- | --- | --- | --- |
|  | 2 | x, y, z | 7.56 | –4.35 | –0.84 | –19.38 | 12.38 | –12.43 |
|  | 2 | – | 10.25 | 0.00 | – | 0.00 | 0.00 | – |
|  | 2 | – | 7.17 | 0.00 | 0.00 | 0.00 | 0.00 | 0.00 |
|  | 4 | –x, y+1/2, –z+1/2 | 16.71 | 0.88 | –0.22 | –3.59 | 0.00 | –2.49 |
|  | 4 | –x, y+1/2, –z+1/2 | 15.62 | –6.76 | –1.34 | –9.56 | 0.00 | –16.37 |
|  | 2 | x, y, z | 10.87 | –0.12 | –0.29 | –5.00 | 1.82 | –3.35 |
|  | 2 | x, y, z | 10.24 | –1.29 | –0.62 | –10.09 | 5.69 | –6.19 |
|  | 2 | – | 7.84 | –1.29 | –0.62 | –10.09 | 5.69 | –6.19 |
|  | 2 | – | 5.44 | –1.29 | –0.62 | –10.09 | 5.69 | –6.19 |
|  | 2 | – | 5.12 | –0.14 | –0.02 | –0.53 | 0.00 | –0.65 |

| **NetE_tot** |  | –14.36 | –4.57 | –68.31 | 31.26 | –53.85 |
| --- | --- | --- | --- | --- | --- | --- |

R is the distance between molecular centroids (mean atomic position) in Å, E_ele = electrostatic energy, E_pol = polarization energy, E_dis = dispersion energy, E_rep = repulsion energy, E_tot = total interaction energy per molecule pairs, and NetE_tot = total energy per energy components.


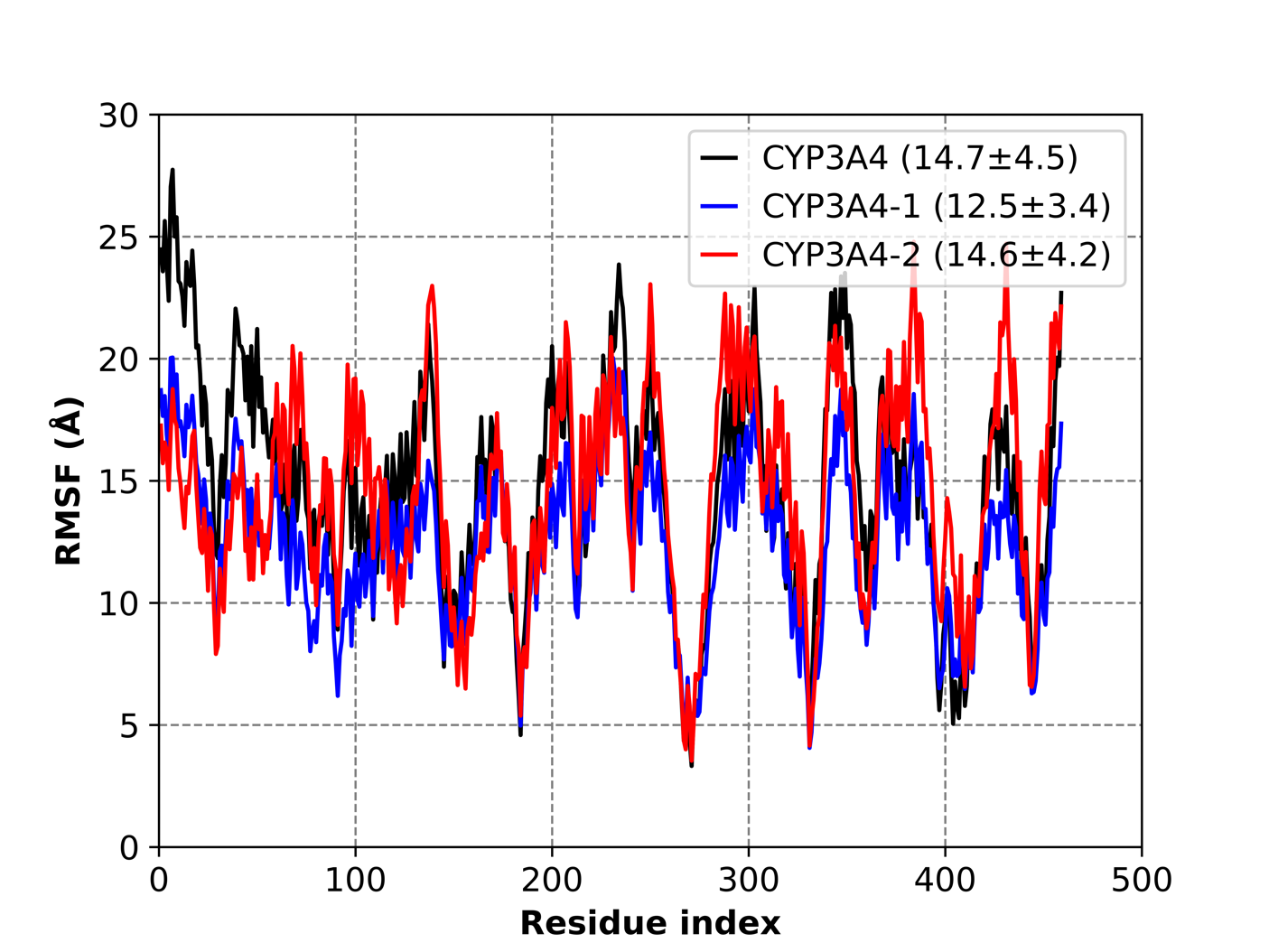


**Figure S5**: All atoms RMSF of CYP3A4 protein per residue for apo and compounds **1** and **2** bound.
